# Revisiting Anti-HCV Seropositivity: Signal Intensity, Diagnostic Uncertainty, and the Limitations of Fixed Cut-Offs

**DOI:** 10.3390/diagnostics16162665

**Published:** 2026-08-21

**Authors:** Baris Otlu, Yusuf Yakupogullari, Elif Seren Tanriverdi

**Affiliations:** Faculty of Medicine, Department of Medical Microbiology, İnönü University, 44280 Malatya, Türkiye; yusufyakoup@yahoo.com (Y.Y.); seren.tanriverdi@inonu.edu.tr (E.S.T.)

**Keywords:** hepatitis C virus, anti-HCV, signal-to-cutoff ratio, diagnostic uncertainty, false-positive results

## Abstract

**Background/Objectives**: A reactive anti-HCV antibody result requires confirmatory testing because seropositivity does not necessarily indicate active hepatitis C virus (HCV) infection. Although anti-HCV signal intensity may provide clinically useful information, it remains underutilized, particularly in low-reactivity ranges where diagnostic uncertainty is substantial. This study evaluated the diagnostic performance of anti-HCV signal-to-cutoff (S/Co) values and aimed to define a clinically meaningful uncertainty interval. **Methods**: In this retrospective study, 565,726 anti-HCV test results were screened, and the analysis was performed within the anti-HCV reactive cohort (≥1.0 S/Co; *n* = 2950), comprising 1581 patients with true HCV infection and 1369 without infection. Infection status was assigned using a uniform virological reference standard, every case being supported by at least one documented positive HCV-RNA PCR result. Receiver operating characteristic (ROC) analysis was used to assess discrimination, and multivariable logistic regression to model the probability of true infection. **Results**: Anti-HCV signal intensity discriminated true infection well across the reactive cohort (AUC 0.991), but less within the diagnostic uncertainty interval (1.00–4.99 S/Co; AUC 0.832), where only 5.7% of reactive results represented true infection. A Youden-optimal threshold of 4.65 S/Co provided >95% sensitivity, specificity and positive predictive value in the reactive cohort. The probability of true infection rose steeply with signal level, and each doubling of the S/Co value increased the odds of infection 23–fold. No threshold within the uncertainty interval raised the positive predictive value above 58.2%. Restricting confirmatory testing to values above 2.50 S/Co would avoid 41.7% of confirmatory tests while missing 1.96% of true infections. **Conclusions**: Anti-HCV signal intensity is strongly associated with the probability of true infection, but a single fixed cut-off is insufficient for clinical interpretation. Graded, signal-based interpretation may improve diagnostic accuracy, clarify low-level reactivity, and reduce unnecessary confirmatory testing.

## 1. Introduction

Hepatitis C virus (HCV) is an enveloped, positive-sense RNA virus that is transmitted predominantly through exposure to infected blood, unsafe medical procedures, and the sharing of syringes among people who inject drugs [1]. The World Health Organization (WHO) estimated that about 50 million people were living with chronic HCV infection, which caused about 250 thousand deaths in 2022 [2]. These deaths are driven primarily by cirrhosis and hepatocellular carcinoma (HCC), and approximately one-fifth of HCC cases are attributable to HCV infection [3]. Historically, HCV-related end-stage liver disease has been one of the leading indications for liver transplantations [4].

Anti-HCV assays are indispensable for community screening, hospital-based case detection, and blood or organ donations. Despite its high analytical sensitivity, anti-HCV testing does not establish whether a reactive result represents a true infection, and current international guidance therefore requires virological confirmation after a reactive antibody result, usually with HCV RNA testing [5]. However, a strong antibody response may be lacking in patients with conditions that impair antibody responses, such as end-stage renal or liver disease, immunosuppression, and HIV coinfection [6,7]. Conversely, false-reactive anti-HCV results occur frequently, and particularly in low-prevalence settings, more false-positive than true-positive results may be detected [8]. In a previous study at our institution, the annual number of anti-HCV-positive patients decreased significantly after 2016, when direct-acting antivirals (DAAs) began to be covered by national health insurance; this decline was concentrated among patients with anti-HCV levels of ≥5 S/Co, with no substantial reduction below this titer [9]. All these findings suggested the need to re-evaluate the ability of anti-HCV results to accurately indicate true HCV infection.

No single antibody-based ELISA or chemiluminescent assay can function as a definitive stand-alone diagnostic test for HCV infection. Most laboratories continue to apply the manufacturer-defined threshold in a binary manner, although the probability of true infection is unlikely to be constant across the entire reactive range. This has led to the non-recommendation of confirmatory testing for low-level positive results (<2.7 S/Co) [10], or to the suggestion of retesting on a different immunoassay platform instead of PCR [11]. Indeed, facility-based data have shown that confirmatory testing was performed for only around half of patients [8,12]. Furthermore, previous studies have proposed optimal S/Co cut-off values for predicting HCV infection, often favoring higher thresholds to improve specificity and positive predictive value. However, most rely exclusively on HCV-RNA PCR as the reference standard, which may not fully capture the biological meaning of anti-HCV reactivity. As HCV-RNA reflects only ongoing viremia, such a standard inevitably classifies individuals with spontaneously resolved infection as false-positives, although these persons carry antibodies produced by a genuine past infection.

In this context, a more nuanced interpretation of anti-HCV reactivity may offer meaningful clinical and laboratory benefits. Rather than treating all reactive results as equivalent, signal thresholds may better stratify the probability of true infection and reduce unnecessary confirmatory testing. Importantly, the interpretation of anti-HCV reactivity in routine practice differs considerably from the idealized condition of a virologically pre-selected cohort. The present study was accordingly designed to reflect a routine clinical diagnostic setting with the data of individuals identified through longitudinal clinical follow-up within the study cohort. By combining receiver operating characteristic analysis with predictive modeling, we aimed to identify clinically informative thresholds that could improve the interpretation of intermediate anti-HCV results, optimize confirmatory test use, and reduce avoidable laboratory burden and patient uncertainty.

## 2. Materials and Methods

### 2.1. Study Design, Setting, and Patient Selection

This study was conducted at İnönü University Turgut Özal Medical Center, a 1500-bed tertiary referral hospital located in eastern Türkiye. The center is a world-ranked facility for liver transplantation and serves as a major referral hub for gastroenterology and hepatology healthcare. Patients with suspected liver disease, including HCV infection, are routinely referred to this center for confirmatory serological and molecular testing, as well as for clinical management and follow up.

Laboratory data were retrospectively obtained from the hospital’s Laboratory Information Management System (LIS). Records of 565,726 anti-HCV antibody tests from 271,421 individuals and 12,266 HCV-RNA PCR assays from 4969 individuals, performed in the Microbiology and Molecular Microbiology laboratories from 1 January 2010 to 31 December 2025, were retrieved. After removal of duplicate entries and repeat tests, a unique 16–year dataset was compiled, yielding 3937 individuals (1.45%) with anti-HCV positivity (≥1 S/Co). This dataset was merged with the records of patients who had undergone HCV-RNA PCR testing and of those with a confirmed diagnosis of HCV infection treated or monitored at our institution. Patients without a definitive clinical diagnosis or with incomplete diagnostic data were excluded. Following this process, a total of 2956 patients were found to be eligible for the study, including 2950 who were anti-HCV-positive and six patients who were anti-HCV-negative but had HCV viremia, and their medical records were analyzed.

Case definition: HCV infection status was assigned using a uniform virological reference standard. Over the 16–year period, 1587 patients were classified as HCV-infected, in every case on the basis of at least one documented positive HCV-RNA PCR result: infection was confirmed by HCV-RNA PCR for 1477 patients in our facility, and a further 110 had been confirmed by HCV-RNA PCR at referring institutions.

Anti-HCV reactive individuals were classified as not infected when no positive HCV-RNA result and no documented diagnosis of HCV infection were present in the institutional record at any point during the study period. Most of these individuals had repeated encounters with our institution over an extended period, and the record captured the complete set of ICD-10 diagnostic codes, comorbidities, and clinic visits assigned to each patient; virological results not available at our centre were not imputed. Consequently, 1369 anti-HCV reactive individuals (≥1 S/Co) were assigned to the control group.

Demographic variables (age, sex), laboratory results, and clinical data were extracted from the database, and all data were anonymized prior to analysis. The study protocol was approved by the İnönü University Health Sciences Scientific Research Ethics Committee (Approval No: 2026/10085).

### 2.2. Laboratory Assays

Anti-HCV Antibody Testing: Serum or plasma samples were analyzed for anti-HCV antibodies using a chemiluminescent microparticle immunoassay (CMIA) with Alinity kits on the Alinity-i macro-ELISA platform (Abbott Laboratories, Abbott Park, IL, USA). Results were expressed as the ratio of the sample relative light unit (RLU) to the cutoff RLU (S/Co). According to the manufacturer’s instructions, an S/Co ratio ≥1.0 was interpreted as positive.

HCV-RNA PCR Testing: Quantitative detection of HCV-RNA in blood samples was performed using a real-time PCR method with artus HCV QS-RGQ V1 kits on the RotorGene-Q RT-qPCR system (Qiagen, Hilden, Germany). All assays were interpreted using internal controls together with positive and negative control samples to ensure analytical validity.

### 2.3. Statistical Analysis

All analyses were performed on a single locked source dataset. Analyses were conducted in Python 3.11 (statsmodels, scikit-learn, pandas, NumPy) and cross-checked in MedCalc v20.0. Statistical significance was set at *p* < 0.05.

Continuous variables (age, anti-HCV S/Co) were non-normally distributed (Kolmogorov–Smirnov and Shapiro–Wilk tests) and are summarized as median and interquartile range (IQR); categorical variables are given as counts and percentages. Intergroup comparisons used the Mann–Whitney U test for continuous variables and the Pearson chi-square test for categorical variables.

Receiver operating characteristic (ROC) analysis was performed for the reactive cohort and for those in the diagnostic uncertainty interval (1.00–4.99 S/Co); the area under the curve (AUC) with 95% confidence intervals was estimated by bootstrap resampling (2000 replicates). Internal validity was assessed by bootstrap optimism correction. Optimal cut-offs were identified using the Youden index (J = sensitivity + specificity − 1). Sensitivity, specificity, PPV, NPV, and likelihood ratios were computed at selected thresholds, each with Wilson 95% confidence intervals. A total of six patients who were anti-HCV negative but HCV-RNA-positive were excluded from the analysis as their anti-HCV antibody levels were below 1.0 S/Co. Because the reactive stratum is a complete census, PPV and NPV were reported as directly observed proportions rather than adjusted to an external seroprevalence. Interval likelihood ratios, which are independent of pre-test probability, were additionally calculated for each anti-HCV signal band to allow application to populations with any local prevalence.

The probability of true infection was modeled by multivariable logistic regression with anti-HCV entered as log_2_(S/Co), reflecting the non-linear relationship between signal intensity and infection probability, and adjusted for age and sex. Model estimates were compared against directly observed band-specific probabilities. Complete and quasi-complete separation were formally excluded (all standard errors < 0.40, with stable convergence).

The effect of restricting confirmatory HCV-RNA testing to values above a given threshold was evaluated within the reactive cohort, reporting for each threshold the proportion of confirmatory tests avoided and the corresponding proportion of true infections missed, with 95% confidence intervals. Because actual unit costs were not incorporated, this is described as a reduction in confirmatory testing rather than a formal cost-effectiveness analysis.

## 3. Results

### 3.1. Analysis of Study Population

Demographic and serological characteristics of the reactive cohort are summarized in Table 1. The HCV-infected patients were older than non-infected individuals (61 vs. 49; *p* < 0.001), the proportion of men slightly higher (55.9% vs. 52.5%; *p* = 0.071) and anti-HCV signal levels were higher among infected patients (14.40 vs. 1.44; *p* < 0.001). Within the diagnostic uncertainty interval, 83 of 1446 reactive individuals (5.7%) had true infection. Anti-HCV levels in this interval were also higher among infected than non-infected individuals (median 3.30, IQR 2.00–4.62, vs. 1.44, IQR 1.15–2.21 S/Co; *p* < 0.001), although the two distributions overlapped substantially. In the reactive cohort, 89.2% of infected patients had anti-HCV values ≥10 S/Co, whereas 99.6% of non-infected individuals had values below 5 S/Co (Table 1).

### 3.2. Diagnostic Performance of Anti-HCV Antibody Test

**Figure 1:**
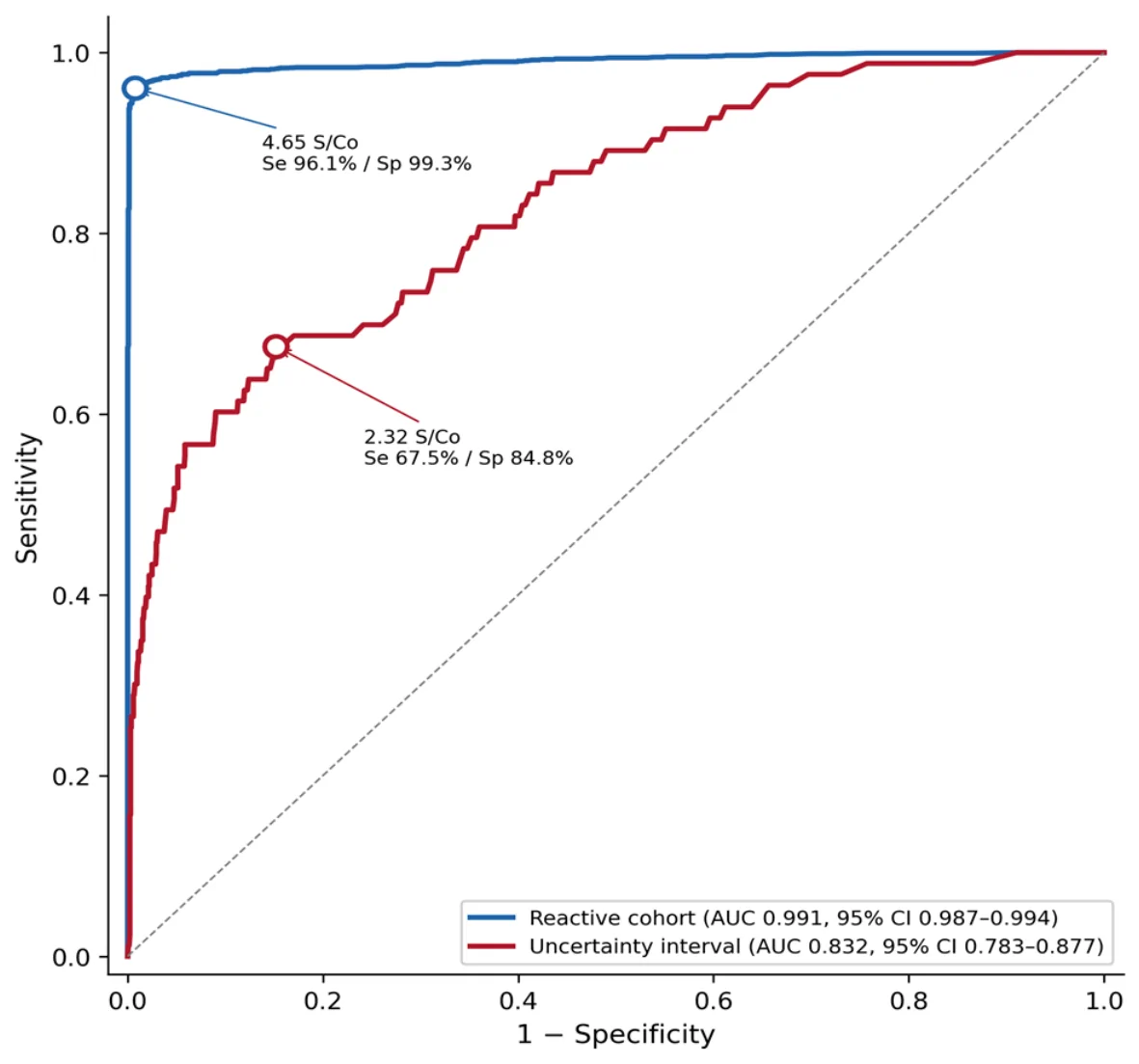
ROC curves of anti-HCV signal intensity for identifying true HCV infection, shown for the reactive cohort (*n* = 2950) and for the diagnostic uncertainty interval (1.00–4.99 S/Co; *n* = 1446). Discrimination was excellent across the reactive cohort (AUC 0.991, 95% CI 0.987–0.994) but substantially lower within the uncertainty interval (AUC 0.832, 95% CI 0.783–0.877). Confidence intervals were estimated by bootstrap resampling (2000 replicates). Circles mark the Youden-optimal cut-offs (4.65 and 2.32 S/Co, respectively) with their corresponding sensitivity and specificity. The dashed diagonal line represents the line of no discrimination (AUC = 0.5), corresponding to the performance of a random classifier.

ROC analysis of the reactive cohort yielded an area under the curve (AUC) of 0.991 (95% CI 0.987–0.994; 2000 bootstrap resamples), and of 0.832 (95% CI 0.783–0.877) for those within the diagnostic uncertainty interval (1.00–4.99 S/Co). Both ROC curves are shown in Figure 1.

Diagnostic performance parameters for the reactive cohort at selected thresholds are presented in Table 2, and those restricted to the diagnostic uncertainty interval in Table 3. The Youden-optimal cut-off for the reactive cohort was 4.65 S/Co, with a sensitivity of 96.1% and a specificity of 99.3%; within the diagnostic uncertainty interval, the Youden-optimal cut-off was 2.32 S/Co, with a sensitivity of 67.5% and a specificity of 84.8%, and the positive predictive value did not exceed 58.2% at any threshold in this interval.

The probability of true infection increased across signal bands; while 2.2% at 1.00–1.99 S/Co and 36.3% at 3.00–4.99 S/Co, it increased to 95.7% (88/92) at 5.00–9.99 S/Co and 99.9% (1410/1412) at ≥10 S/Co. The corresponding interval likelihood ratios are provided in Table 2.

### 3.3. Independent Predictors of True Infection

In multivariable logistic regression with anti-HCV modeled as log_2_(S/Co) and adjusted for age and sex, each doubling of the S/Co value was associated with an odds ratio for true infection of 23.29 (95% CI 17.43–31.13; *p* < 0.001). Age (OR 0.990 per year, 95% CI 0.978–1.002; *p* = 0.099) and sex (OR 1.184; *p* = 0.478) were not independent predictors. Complete and quasi-complete separation were formally excluded, and penalized estimation was not required.

**Figure 2:**
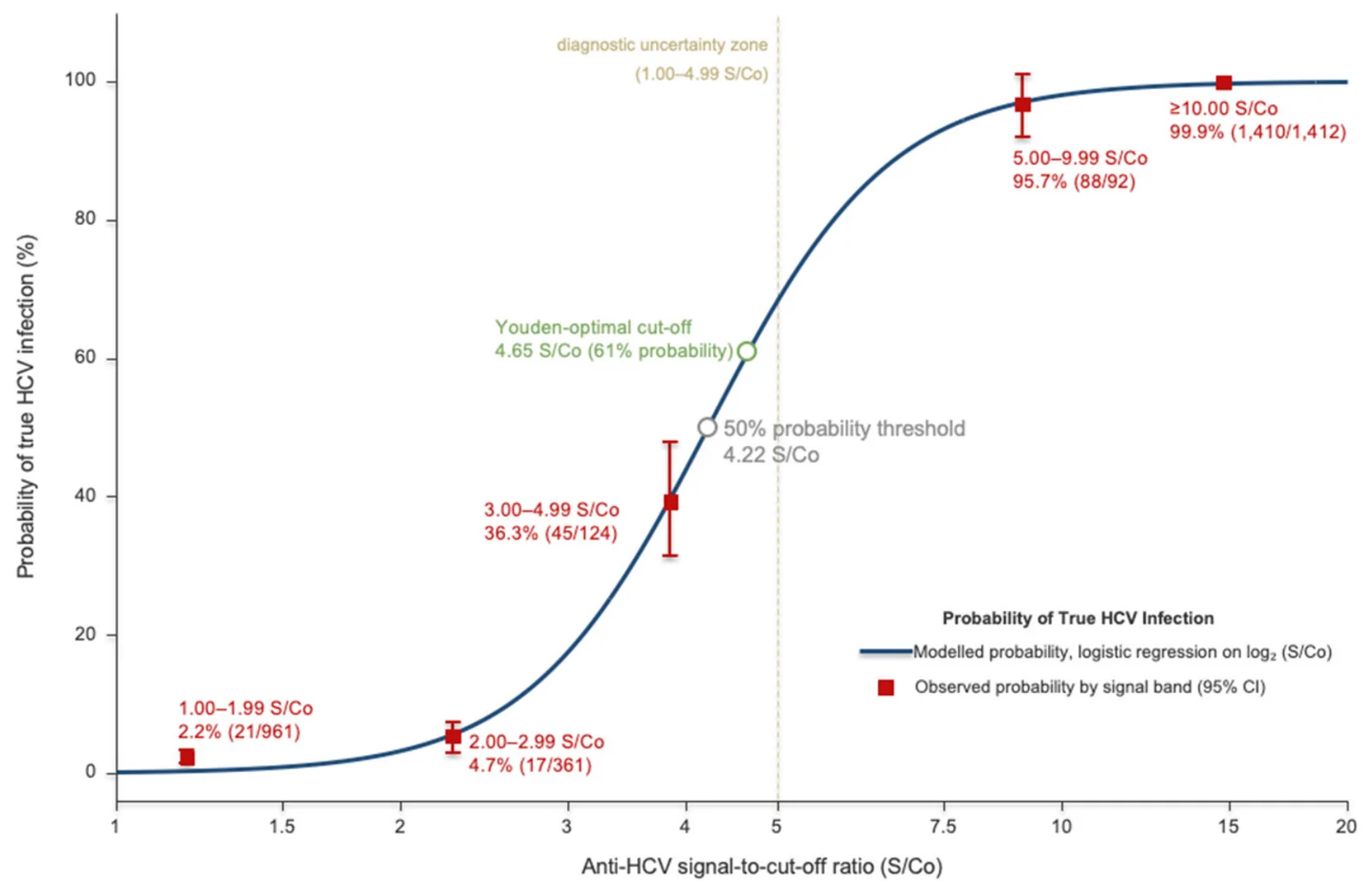
Probability of true HCV infection according to anti-HCV signal intensity in the reactive cohort (*n* = 2950). The solid line shows the probability modeled by multivariable logistic regression on log_2_(S/Co), adjusted for age and sex; squares show the directly observed probability per signal band with Wilson 95% confidence intervals, plotted at each band’s median. The dashed line at 5.00 S/Co separates the diagnostic uncertainty interval (1.00–4.99 S/Co), within which only 5.7% of reactive results represented true infection. Open circles mark the 50% probability threshold (4.22 S/Co) and the Youden-optimal cut-off (4.65 S/Co); the latter corresponds to a post-test probability of 61%, not 50%, because the Youden index weights sensitivity and specificity equally whereas post-test probability depends on the pre-test probability (53.6%).

The modeled probability of true infection followed a sigmoid relationship with signal intensity and reached 50% at 4.22 S/Co. The Youden-optimal cut-off of 4.65 S/Co corresponded to a post-test probability of 61%, given a pre-test probability of 53.6% in the reactive cohort. The modeled probability curve, the observed band-specific probabilities, and the clinically relevant thresholds are shown in Figure 2.

### 3.4. Reduction in Confirmatory Testing

Restricting confirmatory HCV-RNA testing to anti-HCV values above a defined threshold reduced testing volume, with a corresponding proportion of missed infections. Within the reactive cohort, a threshold of 2.50 S/Co avoided 1231 confirmatory tests (41.7%) and missed 1.96% of true infections, a threshold of 3.57 S/Co avoided 1376 tests (46.6%) and missed 2.97%, and a threshold of 5.00 S/Co avoided 1446 tests (49.0%) and missed 5.25%. Because actual unit costs were not incorporated, these figures represent reductions in confirmatory testing rather than a formal cost-effectiveness analysis.

## 4. Discussion

Anti-HCV antibody testing is optimized to maximize sensitivity, and at the conventional 1.0 S/Co threshold it detects almost all true infections. This design is essential in clinical contexts where missing an infection would have serious consequences. In populations with low disease prevalence, however, and particularly in settings such as ours where anti-HCV testing is incorporated into routine screening panels, this strategy generates a large volume of low-level reactive results that do not correspond to true infection and that trigger extensive confirmatory or reflex testing. Therefore, in this study, we examined how the probability of true HCV infection varies with anti-HCV signal intensity across a large reactive cohort, and we found that a single reactive result carries a diagnostic weight that ranges from negligible to near-certain depending on its signal level. The 16-year span of the dataset provided a relatively large number of patients for whom sufficient diagnostic and follow-up information was available, allowing infection status to be established with confidence rather than inferred from a single laboratory value. Because the study was conducted at a regional gastroenterology and liver-transplantation referral centre, the reactive cohort arose from a broad range of clinical contexts in which anti-HCV testing is performed, including transplant candidates and recipients, immunosuppressed patients, and individuals referred with an established diagnosis, rather than from a narrow, virologically pre-selected population. Together, these features allowed the use of a uniform virological reference standard and the analysis of the reactive population as a complete census.

In our study, patients with confirmed HCV infection were significantly older than non-infected individuals (Table 1). This finding is consistent with the cumulative acquisition of HCV over a lifetime and with our two previous long-term analyses: since the reimbursement of direct-acting antivirals for HCV in 2016 and the national HBV vaccination campaigns, new infections have declined for both viruses, so that the previously infected cohort ages progressively while few younger patients enter it [9,13]. The advancing age is associated with weakening humoral responses [14,15], and antibody titers may decline over time after spontaneous clearance [16,17]; both processes add low-level reactive results that represent past rather than current infection, reinforcing the ambiguity of the low-signal range. The relationship between signal intensity and the likelihood of true infection showed a clear pattern. Namely, in the 1.00–1.99 S/Co range, only about 2% of reactive results reflected genuine infection, whereas this proportion rose approximately 16 –fold in the 3.00–4.99 range, and exceeded 95% once the signal reached 5 S/Co or above (Figure 2). In other words, a reactive result near the assay cutoff was, in this cohort, far more likely to be a non-specific signal than a marker of active infection. This pattern indicates that each reactive result carries a distinct diagnostic weight that is obscured when the result is interpreted in a binary manner. In multivariable analysis, each doubling of the S/Co value was associated with a 23–fold increase in the odds of true infection, independent of age and sex.

This divergence in diagnostic value across the signal range was reflected in the ROC analyses (Figure 1). For the reactive cohort, anti-HCV signal intensity discriminated true infection almost perfectly (AUC 0.991), confirming that the S/Co value is a powerful quantitative marker. However, discrimination fell substantially to an AUC of 0.832 when the analysis was confined to the interval of 1.00–4.99 S/Co. The contrast between these two curves visualizes the central problem of low-level reactivity where clinical decisions are most difficult.

Our analysis showed that diagnostic performance parameters shift sharply with the chosen threshold. At the conventional 1.0 S/Co cutoff, which by definition includes the entire reactive cohort, all 1369 non-infected reactive individuals are classified as positive; this row represents a reference baseline rather than a diagnostic performance estimate. Raising the decision threshold to the Youden-optimal 4.65 S/Co increases specificity to 99.3% and reduces the number of false positives from 1369 to 10 while sensitivity declines only modestly to 96.1%. The positive predictive value rises in parallel from 53.6% to 99.3% (Table 2). This asymmetry, in which a large gain in specificity costs only a few percentage points of sensitivity, is the practical basis for signal-based interpretation and explains why the great majority of confirmatory tests become unnecessary in the lowest part of the reactive range. Within the diagnostic uncertainty interval, the same threshold dependence is present but never reaches a level sufficient for standalone diagnosis. At the Youden-optimal cutoff of 2.32 S/Co, the best available balance yielded a sensitivity of only 67.5% and a positive predictive value of 21.3% (Table 3). No threshold within this interval simultaneously maintained acceptable sensitivity and predictive value.

Previous studies have generally reported slightly higher optimal cut-offs for the anti-HCV signal-to-cutoff ratio, ranging from 4.32 to 10.86 (median 6), with positive predictive values from 44.2% to 87.4% [8,18,19,20,21,22,23]. Most of these studies, like ours, used HCV-RNA PCR as the reference standard. This approach identifies current viremia rather than the entire biological meaning of anti-HCV reactivity: approximately 15–45% of infected individuals undergo spontaneous viral clearance and continue to carry antibodies produced by a genuine past infection [24]. Such individuals cannot be captured by a PCR and are necessarily counted as non-infected, so that the probabilities reported here, as in comparable studies, should be read as probabilities of PCR-confirmable infection rather than of any previous exposure to HCV. The higher predictive values we observed follow from how the compared groups are defined rather than from assay behaviour: where the target condition is current viraemia, patients with treated or spontaneously cleared infection retain high antibody titres and are counted among the false positives, whereas our non-infected group contains only individuals with no documented evidence of HCV infection at any time, and their values are concentrated at the bottom of the reactive range (99.6% below 5.00 S/Co). The case group also differs: sixteen years of records from a regional referral centre for gastroenterology, transplantation, and oncology accumulate patients with long-standing chronic infection, who are older and show a correspondingly mature and uniform antibody response, whereas community-based and blood-donor cohorts span wider age ranges and shorter disease durations. This is also what gives the present dataset its value: in a region of very low HCV frequency, a reactive population of this size and diagnostic completeness could not readily be assembled other than through the longitudinal records of a referral centre, and it is in such centres—transplantation, dialysis, and oncology services—that the interpretation of an intermediate anti-HCV result carries the most immediate consequences for patient management.

The probability model integrates these observations into a single continuous relationship. Rather than a step change at any particular cutoff, the modeled probability of true infection rose sigmoidally with signal intensity, crossing 50% at 4.22 S/Co (Figure 2). An important nuance emerged from comparing this probability threshold with the ROC-derived cutoff: although the Youden-optimal value (4.65 S/Co) is close numerically, it corresponds to a post-test probability of 61% rather than 50%, because the Youden index balances sensitivity and specificity equally, whereas post-test probability also depends on the pre-test probability of the tested population. Discrimination-based and probability-based thresholds therefore answer different questions and should be applied as complementary tools rather than as a single universal cutoff.

These relationships have an apparent operational consequence for confirmatory testing. Because the overwhelming majority of low-signal reactive results are not true infections, restricting reflex HCV-RNA testing to values above a chosen threshold can substantially reduce testing volume at a defined and transparent cost. In our cohort, even a threshold of 2.50 S/Co would avoid 41.7% of confirmatory tests, representing a substantial saving for routine clinical diagnosis. Nevertheless, it would still miss approximately 2% of true infections, which could be a critical problem in certain clinical situations. This missed fraction is concentrated in the lower part of the diagnostic uncertainty interval and represents the most important challenge to the otherwise consistent relationship we observed between increasing signal intensity and true infection. At the same time, it constitutes a strong argument that anti-HCV assays still need to be refined specifically within this range in order to improve their sensitivity and specificity. Furthermore, each laboratory should select an operating point according to local prevalence, resources, and the clinical acceptability of a missed infection, and the accompanying interval likelihood ratios allow these observations to be applied at different local pre-test probabilities.

Signal-based interpretation is intended to support, and not to replace, virological confirmation. In our series, six individuals with a positive HCV-RNA result had anti-HCV values below the reactivity threshold; all were solid-organ transplant recipients receiving immunosuppressive therapy, and five of the six had values immediately below the cut-off (0.81–0.96 S/Co). Although such cases were exceedingly rare, representing 0.38% of all confirmed infections over 16 years, they indicate that a non-reactive or low-level anti-HCV result does not exclude infection in profoundly immunosuppressed patients, in the early serological window, or in dialysis and transplant populations [6,7]. Confirmatory HCV-RNA testing therefore remains essential in these groups regardless of signal intensity.

This study has several limitations. It is a retrospective, single-centre analysis, and infection status could not be established through a uniform prospective protocol. All anti-HCV measurements were obtained with a single chemiluminescent immunoassay platform, so the thresholds reported here should be validated before being applied to other platforms. The study was conducted in a region of relatively low HCV frequency, which increases the proportion of low-level and false-reactive results. Infection was defined by a documented positive HCV-RNA result, so individuals with spontaneously resolved infection are not represented among the cases; because they retain anti-HCV antibodies, their assignment to the non-infected group would bias the band-specific probabilities downward. Confirmatory testing is also not applied uniformly across the signal range in routine practice, and residual differential verification would tend to accentuate the separation observed at the lower end of the reactive range.

These findings come from a referral cohort in which chronic HCV infection is concentrated, so the exact thresholds may not transfer unchanged to community screening or blood-donor settings. The shape of the relationship, however, does not depend on the setting: the probability of true infection rises continuously with signal intensity, and the 1.00–4.99 S/Co interval cannot be resolved by signal intensity alone—a limitation unlikely to be smaller where antibody responses are more heterogeneous—while the interval likelihood ratios provided allow the findings to be applied at other pre-test probabilities.

## 5. Conclusions

Anti-HCV signal intensity is a strong quantitative indicator of true HCV infection across most of its range, but its diagnostic value collapses precisely within the low-to-intermediate interval where interpretation is most difficult. Treating all reactive results as equivalent therefore misrepresents their very different diagnostic weights. A graded, probability-based interpretation of the S/Co value, combined with selective confirmatory testing and, where necessary, longitudinal clinical assessment, offers a more accurate and more efficient approach to anti-HCV reactivity than a single fixed threshold.

## Figures and Tables

**Table 1:** Demographic and serological characteristics of the study population (*n* = 2950).

Characteristics	HCV Patients (*n* = 1581)	No HCV Infection (*n* = 1369)	*p*
Age (years)			
Median (IQR)	61 (51–68)	49 (31–64)	<0.001 *
Gender			
Male, *n* (%)	884 (55.9%)	719 (52.5%)	0.071 **
Female, *n* (%)	697 (44.1%)	650 (47.5%)	
Anti-HCV (S/Co), Median (IQR)	14.40 (12.43–16.44)	1.44 (1.15–2.21)	<0.001 *
Anti-HCV Category			
1.00–1.99 S/Co, *n* (%)	21 (1.3)	940 (68.7)	
2.00–2.99 S/Co, *n* (%)	17 (1.1)	344 (25.1)	
3.00–4.99 S/Co, *n* (%)	45 (2.8)	79 (5.8)	
5.00–9.99 S/Co, *n* (%)	88 (5.6)	4 (0.3)	
≥10.00 S/Co, *n* (%)	1410 (89.2)	2 (0.1)	

* Mann–Whitney U test; ** Pearson chi-square test. IQR, interquartile range; S/Co, signal-to-cut-off ratio.

**Table 2:** Diagnostic performance of anti-HCV S/Co at selected thresholds within the reactive cohort (*n* = 2950).

Cut-Off	Sensitivity, % (95% CI)	Specificity, % (95% CI)	PPV, % (95% CI)	NPV, % (95% CI)	J	LR+	TP	FP	TN	FN
1.00	100.00 (99.8–100.0)	0.00 (0.0–0.3)	53.6 (51.8–55.4)	—	0.000	1.00	1581	1369	0	0
2.50	98.04 (97.2–98.6)	87.66 (85.8–89.3)	90.2 (88.7–91.5)	97.5 (96.4–98.2)	0.857	7.94	1550	169	1200	31
3.57	97.03 (96.1–97.8)	97.08 (96.0–97.8)	97.5 (96.6–98.1)	96.6 (95.5–97.4)	0.941	33.21	1534	40	1329	47
4.65 *	**96.08 (95.0–96.9)**	**99.27 (98.7–99.6)**	**99.3 (98.8–99.6)**	**95.6 (94.4–96.6)**	**0.953**	**131.5**	**1519**	**10**	**1359**	**62**
5.00	94.75 (93.5–95.7)	99.56 (99.0–99.8)	99.6 (99.1–99.8)	93.3 (92.9–95.3)	0.943	216.2	1498	6	1363	83
10.00	89.18 (87.6–90.6)	99.85 (99.5–100.0)	99.9 (99.5–100.0)	88.9 (87.2–90.4)	0.890	610.5	1410	2	1367	171

* Youden-optimal threshold. All predictive values are observed proportions within the reactive census and are not adjusted to an external prevalence. Wilson 95% confidence intervals. **J**, Youden index; **LR+**, positive likelihood ratio; **PPV**, positive predictive value; **NPV**, negative predictive value; **TP**, true positive; **FP**, false positive; **TN**, true negative; **FN**, false negative. At the 1.0 S/Co threshold, sensitivity is 100% and specificity 0% by construction, because the analysis was restricted to the anti-HCV reactive cohort (≥1.0 S/Co); this row represents a reference baseline for the entire reactive population rather than a diagnostic performance estimate. Six viremic individuals with anti-HCV below 1.0 S/Co were excluded from this analysis.

**Table 3:** Diagnostic performance of anti-HCV S/Co at selected thresholds within the diagnostic uncertainty interval (1.00–4.99 S/Co; n= 1446).

Cut-Off	Sensitivity, % (95% CI)	Specificity, % (95% CI)	PPV, % (95% CI)	NPV, % (95% CI)	J	LR+	TP	FP	TN	FN
1.00	100.00 (95.6–100.0)	0.00 (0.0–0.3)	5.74 (4.7–7.1)	—	0.000	—	83	1363	0	0
1.50	86.75 (77.8–92.4)	52.68 (50.0–55.3)	10.04 (8.1–12.5)	98.49 (97.3–99.2)	0.394	1.83	0.252	72	645	718
2.00	74.70 (64.4–82.8)	68.97 (66.5–71.4)	12.78 (10.1–16.1)	97.81 (96.7–98.6)	0.437	2.41	0.367	62	423	940
2.32 *	67.47 (56.8–76.6)	84.81 (82.8–86.6)	21.29 (16.8–26.6)	97.72 (96.7–98.4)	0.523	4.44	0.384	56	207	1156
2.50	62.65 (51.9–72.3)	88.04 (86.2–89.7)	24.19 (18.9–30.3)	97.48 (96.4–98.2)	0.507	5.24	0.424	52	163	1200
3.00	54.22 (43.5–64.5)	94.20 (92.8–95.3)	36.29 (28.4–45.0)	97.13 (96.1–97.9)	0.484	9.35	0.486	45	79	1284
4.00	38.55 (28.8–49.3)	98.31 (97.5–98.9)	58.18 (45.0–70.3)	96.33 (95.2–97.2)	0.369	22.85	0.625	32	23	1340

* Youden-optimal cut-off within this interval (Jmax = 0.523); AUC = 0.832 (95% CI 0.783–0.877; 2000 bootstrap resamples). Wilson 95% confidence intervals. **J**, Youden index; **LR+**, positive likelihood ratio; **PPV**, positive predictive value; **NPV**, negative predictive value; **TP**, true positive; **FP**, false positive; **TN**, true negative; **FN**, false negative. At the 1.0 S/Co threshold, sensitivity is 100% and specificity 0% by construction, because the analysis was restricted to the anti-HCV reactive cohort (≥1.0 S/Co); this row represents a reference baseline for the entire reactive population rather than a diagnostic performance estimate. Six viremic individuals with anti-HCV below 1.0 S/Co were excluded from this analysis.

## Data Availability

The datasets generated and/or analyzed during the current study are available from the corresponding author upon reasonable request.

## Abbreviations

The following abbreviations are used in this manuscript:

AUC	Area under the curve
CI	Confidence interval
CMIA	Chemiluminescent microparticle immunoassay
DAAs	Direct-acting antivirals
ELISA	Enzyme-linked immunosorbent assay
FN	False negative
FP	False positive
HCC	Hepatocellular carcinoma
HCV	Hepatitis C virus
HIV	Human immunodeficiency virus
IQR	Interquartile range
LIS	Laboratory Information Management System
